# From *Drosophila* development to adult: clues to Notch function in long-term memory

**DOI:** 10.3389/fncel.2013.00222

**Published:** 2013-11-21

**Authors:** Jiabin Zhang, Jerry C. P. Yin, Cedric S. Wesley

**Affiliations:** ^1^Neuroscience Training Program, University of Wisconsin-MadisonMadison, WI, USA; ^2^Department of Genetics and Medical Genetics, University of Wisconsin-MadisonMadison, WI, USA; ^3^Department of Neurology, University of Wisconsin-Madison, Madison, WI, USA

**Keywords:** notch, LTM, CREB, PKC, F-actin, IκB, Wnt, neurodegeneration

## Abstract

Notch is a cell surface receptor that is well known to mediate inter-cellular communication during animal development. Data in the field indicate that it is also involved in the formation of long-term memory (LTM) in the fully developed adults and in memory loss upon neurodegeneration. Our studies in the model organism *Drosophila* reveal that a non-canonical Notch-protein kinase C activity that plays critical roles in embryonic development also regulates cyclic-AMP response element binding protein during LTM formation in adults. Here we present a perspective on how the various known features of Notch function relate to LTM formation and how they might interface with elements of Wingless/Wnt signaling in this process.

## NOTCH IN MEMORY FORMATION

Notch is a cell surface receptor that mediates intercellular communications through physical contact. It is well known for its roles in the regulation of a wide array of biological processes, in animals from hydra to humans. Much data in the field relates to its roles in development and cell differentiation but there is significant evidence that Notch also plays critical roles in numerous adult processes (Ables et al., 2011). Here we focus on its role in long-term memory (LTM) formation.

The initial study that reported a role for Notch in LTM examined mice using the water maze paradigm and found memory deficits in the heterozygous *Notch1*^+/-^ and the *RBP-j*^+/-^ mice (Costa et al., 2003). *RBP-j* gene is a critical component of the canonical Notch signaling pathway. A caveat with this study is that developmental effects of heterozygosity were not controlled. Another study that used a conditional system found that postnatal knockout of *RBP-j* in the excitatory neurons did not impair memory formation (Sato et al., 2012). This result raises questions about the role of canonical Notch signaling in the process. Canonical Notch signaling is activated upon ligand binding and results in the Notch intracellular domain being released from the plasma membrane (NICD). NICD is transported to the nucleus where it associates with RBP-j (the DNA binding factor) and up-regulates transcription of target genes. Since NICD requires RBP-j to bind DNA, the *RBP-j* conditional knock out data appears to rule out the involvement of canonical Notch signaling in LTM formation. It could also rule out any Notch-independent RBP-j activity. Of course, it will not rule out these possibilities, if an unknown paralog fills in to perform the role of *RBP-j*. However, this study does not rule out the involvement of Notch. Although there is a reduction in Notch1 protein level in *RBP-j* knockout tissues, a significant amount persists. It raises the possibility that Notch function in memory formation involves one of the non-canonical signaling mechanisms (Heitzler, 2010; Guruharsha et al., 2012).

Two studies in *Drosophila* adults that used conditional Notch mutants and inducible transgenes clearly demonstrate a role for Notch in memory formation (Ge et al., 2004; Presente et al., 2004). These studies used the olfaction-based, Pavlovian paradigm and showed that Notch is required for LTM but not learning. Amazingly, when the full length Notch protein (NFull) was expressed before training, a single training was sufficient to form significant memory instead of 10 required in control flies (Ge et al., 2004). Similar experiments with *Suppressor of Hairless* [*Su(H)*], the *Drosophila* homolog of *RBP-j*, show that it is also important for LTM (Song et al., 2009). This report shows that LTM is specifically blocked in *Su(H)* mutants and the expression of the wild-type Su(H) protein in mushroom bodies, the key brain region for *Drosophila* LTM, is sufficient to rescue the memory defect. Interestingly, the study also showed that over-expression of Su(H) protein in the wild-type background caused LTM defects (Song et al., 2009). Another study has identified the homotypic cell adhesion molecule Klingon as functioning downstream of Notch in LTM (Matsuno et al., 2009), but it’s not clear whether it is regulated by NICD. Thus, data from both mice and *Drosophila* raise doubts about the involvement of canonical Notch signaling in LTM. The confounding data relate to RBP-j/Su(H) knockout and over-expression.

The relationship between Notch and RBP-j/Su(H) is not simple. Su(H) knockout in *Drosophila* results in the loss of not only NICD but also NFull expression (Kidd et al., 1998; Lecourtois and Schweisguth, 1998; Wesley and Mok, 2003). The Notch receptors that are stable in the absence of Su(H) are the naturally produced, truncated Notch receptors lacking the carboxyl terminal ubiquitination, transcription activation domain, and PEST sequences (Wesley and Mok, 2003). On the other hand, over-expression of Su(H) results in increased nuclear localization of NICD that is in the background (Kidd et al., 1998). Similar relationships between RBP-j and the full length Notch1 protein and NICD can be found in mammals as well (Schroeter et al., 1998; Sato et al., 2012). In addition, Notch and Su(H) display a stoichiometric relationship that appears to determine whether Su(H), NICD, or both are retained in the cytoplasm or translocated to the nucleus (Gho et al., 1996; Kidd et al., 1998). A further complicating matter is that NICD expression from a transgene in the wild-type background suppresses the cell surface expression of NFull produced from the endogenous *Notch* gene, possibly due to titration of Su(H) (Bardot et al., 2005). Incidentally, this observation also implies that transgenic expression of NICD in the wild-type background while reproducing *bona fide* functions of endogenously produced NICD could also manifest additional effects linked to the loss of non-canonical NFull functions at the cell surface. Thus, manipulation of RBP-j/Su(H) may not be the best way to determine whether the canonical or a non-canonical Notch signaling activity is involved in a process. Since a vast amount of data from worms to humans indicates that transgenic expression of NICD reproduces functions that are based on canonical Notch signaling, the best approach could be to use NICD for determining if canonical signaling is involved and explore other non-canonical Notch mechanisms if it is not.

## NON-CANONICAL NOTCH SIGNALING MECHANISMS IN D*rosophila* DEVELOPMENT

Since much information on Notch function in LTM formation is from *Drosophila*, we will restrict ourselves to this model organism.

A non-canonical Notch mechanism is known to function during the development of a class of adult sensory bristles called microchaetae. The development of these bristles is suppressed by a collection of *Notch* alleles (called *mcd* alleles) with mutations that delete the carboxy-terminal portion of the Notch protein (thereby the transcriptional activation domain and the PEST sequences). *mcd* alleles signal through a poorly understood signaling mechanism that interfaces with the Wingless/Wnt pathway (Ramain et al., 2001). However, as this signaling persists in the *Su(H)* knock out background it is not clear whether it is based on NFull or the naturally produced truncated Notch receptors lacking the carboxyl terminus (that would be stable in the absence of Su(H)). Regardless, this non-canonical signaling is closely linked to another non-canonical Notch activity reported, one that is independent of Delta or Serrate ligands and degrades Armadillo/β-Catenin that is a critical component of the Wingless/Wnt pathway (Hayward et al., 2008). As Armadillo/β-Catenin degradation would suppress LTM formation (Tan et al., 2013), it is unlikely that either of these two non-canonical mechanisms is involved in the Notch-mediated *enhancement* of LTM formation.

A non-canonical Notch activity involving Abl kinase regulates Axon pathfinding during embryogenesis. This activity is also shown to persist in the *Su(H)* knock out background (Le Gall et al., 2008). Furthermore, the developing neurons of the central nervous system in the embryos express very high levels of a naturally produced truncated Notch molecule that lacks most of the Notch intracellular domain (LeComte et al., 2006). Thus, it is not clear whether this non-canonical Notch activity is based on NFull or a naturally produced truncated Notch receptor. However, as there is very little evidence that Abl kinase promotes LTM, we do not discuss this Notch activity any further.

We recently discovered another non-canonical Notch function that is involved in dorsal closure and dorso-ventral axis formation in embryos. Dorsal closure is a zipper-like process driven by F-actin dynamics that remodels and mobilizes lateral epithelial cells to close the dorsal “hole” being created by the apoptosing extra-embryonic amnioserosal cells (Harden, 2002). Notch involvement in dorsal closure was reported previously but the underlying signaling mechanism and its target were obscure (Zecchini et al., 1999). The dorso-ventral axis is established by the opposing gradients of Toll/Dorsal and Dpp signaling. Dorsal is the *Drosophila* homolog of NFκB and Dpp is the *Drosophila* homolog of TGFβ/Bone Morphogenetic Protein. The newly discovered non-canonical Notch activity was found to up-regulate the level of F-actin and promote the formation of the longitudinal F-actin cables during dorsal closure (Wesley et al., 2011). During dorso-ventral axis formation, it was found to up-regulate the level of a phosphorylated form of Cactus, the *Drosophila* homolog of IκB, that is a negative regulator of Toll/Dorsal (NFκB) signaling (Tremmel et al., 2013).

Some important features of the new non-canonical Notch signaling are identified (Wesley et al., 2011; Tremmel et al., 2013). This signaling is based on NFull, is activated soon after ligand binding, and involves the activity of Pkc98E, a *Drosophila* homolog of the novel isoform of protein kinase C (PKC). Treatment of Notch expressing cells with diacyl glycerol (DAG) analog elicits the same response as ligand treatment. DAG analog treatment is known to result in plasma membrane localization and activation of PKC (Akiba et al., 2002). As activated NFull, PKC, Cactus, and F-actin exhibit significant overlap in their expression at the cell surface (Wesley et al., 2011; Tremmel et al., 2013), it is possible that NFull activation promotes interactions among these proteins. This possibility is supported by the information that (1) Cactus was initially isolated in a yeast two-hybrid system screen using the Notch ankyrin repeats as the bait (Kidd, 1992), (2) a mammalian homolog of *Drosophila* Pkc98E associates with F-actin during neuronal differentiation (Prekeris et al., 1998; Zeidman et al., 2002), and Pkc98E contains a domain similar to the Notch ankyrin repeats (Tremmel et al., 2013). Apparently, the non-canonical NFull-PKC signaling competes with canonical Notch signaling for NFull: suppression of Pkc98E expression while reproducing mutant phenotypes related to the loss of NFull-PKC activity also results in mutant phenotypes related to increased canonical Notch signaling. Finally, our studies show that the *Drosophila* embryo can be divided into distinct zones based on whether the canonical Notch signaling is up-regulated (e.g., ventral region) or the non-canonical NFull-PKC signaling is up-regulated (e.g., lateral regions; Wesley et al., 2011; Tremmel et al., 2013). Since Notch activities are importantly regulated at the levels of trafficking and recycling to the cell surface (Reichardt and Knoblich, 2013), it is possible that some of these regulations are involved in modulating the relative levels of Notch signaling activities at the cell surface and in the nucleus.

## NFULL-PKC ACTIVITY IN MEMORY FORMATION IN D*rosophila* ADULTS

Cyclic-AMP response element binding protein (CREB) is a transcriptional factor that plays pivotal roles in intrinsic and synaptic plasticity during LTM formation (Yin et al., 1995; Benito and Barco, 2010; Chen et al., 2012; Hirano et al., 2013). CREB over-expression prior to olfaction-based training was also found to reduce from 10 to 1 the number of training required for forming LTM (Yin et al., 1995; Tubon et al., 2013). We studied Notch and CREB together in memory formation in adult flies using temperature-sensitive conditional and inducible alleles and transgenes. We found that NFull-PKC activity up-regulates the level of a hyper-phosphorylated form of CREB (hyper-PO4 CREB; Zhang et al., 2013). Remarkably, the experimental details either in adult flies or in cultured cells were similar to the regulation of P-Cactus. Incidentally, Cactus and CREB share functionally related phosphorylation sites (Taylor et al., 2000). Hyper-PO4 CREB is cytoplasmic (just as P-Cactus) and one of the residues phosphorylated is Serine 231. This Serine is equivalent to Serine 133 in mammalian CREB, the phosphorylation of which is shown to be important for LTM in mammals (Gonzalez and Montminy, 1989; Silva et al., 1998).

We also found an intriguing feature: a single pulse of Notch activity triggers an ultradian oscillation of hyper-PO4 CREB level that is linked to accumulation of nuclear CREB isoform. Wild-type flies also show robust hyper-PO4 CREB oscillation during daytime and after olfaction-based training for memory formation. These observations raise the possibility that the frequency and the amplitude of hyper-PO4 CREB ultradian oscillation are used for repeating the strength of the initial LTM-forming stimulus. Such repetition might be useful for memory consolidation and for identifying the LTM-forming stimulus. It could be also used to store information as wave tracks in the brain that differ in their ability for persistance or reactivation, akin to the way amplitude and frequency of electromagnetic waves are used to convey, store, and retrieve information.

**FIGURE 1:**
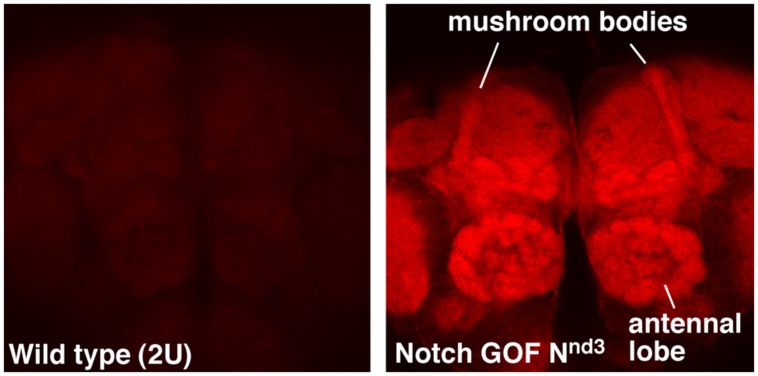
**F-actin is enriched in specific regions of the fly brain immediately after Notch activation.** Mushroom bodies and antennal lobes where Notch is known to function are labeled. N^nd3^ is the same temperature-sensitive *Notch* GOF allele used in Zhang et al. (2013). The wildtype (2U) and N^nd3^ flies were incubated at 30°C for 30 min to activate Notch and the brains were dissected and fixed immediately (see Zhang et al., 2013 for details). Dissected brains were processed identically and F-actin detected using Alexa 568-Phalloidin.

NFull-PKC activity up-regulates not only hyper-PO4 CREB but also F-actin in the adult brains, with a much higher level accumulating in the mushroom bodies and antennal lobes (**Figure 1**). Mushroom bodies are the primary centers for LTM formation and Notch and CREB functions are required there (Presente et al., 2004; Yu et al., 2006; Hirano et al., 2013). Antennal lobe is also shown to require Notch function, in olfaction stimulation (Lieber et al., 2011). An increase in F-actin level has been reported in association with forgetting in *Drosophila* (Davis, 2010; Shuai et al., 2010). However, the forgetting mechanism appears to be independent of cyclic-AMP and CREB pathways. Thus, the Notch-mediated up-regulation of F-actin might be involved in a different F-actin process that promotes LTM formation. That a single pathway could regulate CREB and F-actin could be significant since F-actin dynamics are known to play diverse roles in neuronal functions, from modification of synapses to molecular transport.

## POTENTIAL FOR CROSSTALK WITH WINGLESS/Wnt SIGNALING

**FIGURE 2:**
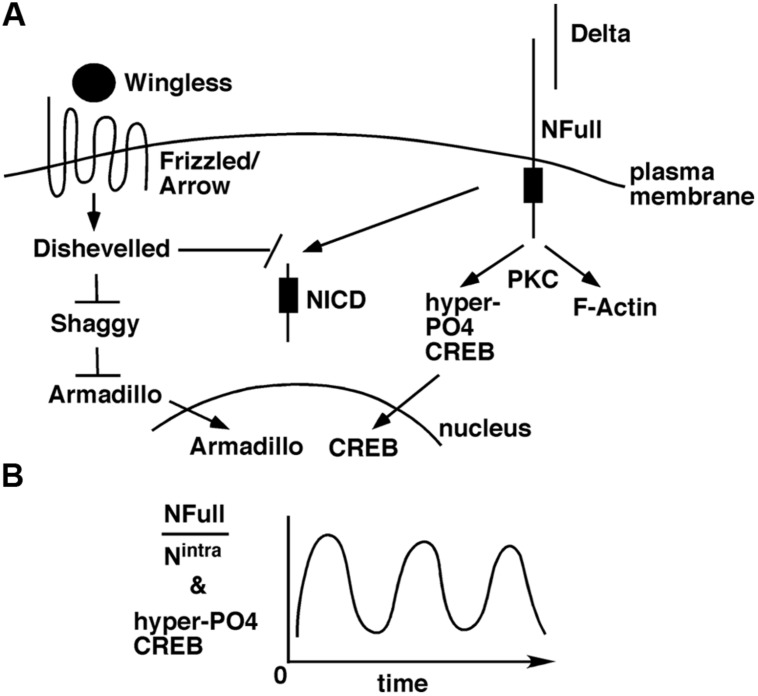
**A model for how the non-canonical NFull-PKC activity, canonical Notch signaling, and Wingless/Wnt signaling might funtion during LTM formation.** **(A)** LTM forming stimulus activates Delta and Wingless that in turn activate NFull and Dfrizzled/Arrow receptors, respectively, and result in nuclear activities of Armadillo and CREB. Wingless/Wnt signaling might also block NICD activity, which might be important for modulating the frequency of hyper-P04 CREB oscillation. **(B)** Ultradian oscillation of hyper-P04 CREB might be genereted by the periodic fluctuation in the ratio of NFull and NICD levels.

The Wingless/Wnt pathway and the Notch pathway often function in the same contexts, in development (Andersen et al., 2012) and adults (Inestrosa and Arenas, 2010; Niehrs, 2012). The target of Wingless/Wnt signaling is the transcriptional activity of Armadillo/β-Catenin in the nucleus. In the absence of this signaling, Armadillo/β-Catenin is targeted for degradation by Shaggy/GSK3 kinase. When Wingless/Wnt binds its receptor complex composed of Frizzled and Lrp/Arrow, the intracellular protein Disheveled is activated, which in turn blocks the Shaggy/GSK3 activity. As a consequence, Armadillo/β-Catenin is stabilized and translocated to the nucleus for activation of target genes. A few weeks ago, an exciting finding was reported: suppression of Wingless, Armadillo/β-Catenin, or Arrow expression in the mushroom bodies suppresses LTM formation in adult flies (Tan et al., 2013). An earlier study has shown that Disheveled can bind to the Notch intracellular domain and inhibit canonical Notch signaling (Axelrod et al., 1996). Taken together, an interesting possibility arises. A stimulus for LTM formation results in the activation of Wingless/Wnt and Delta ligands. These ligands activate Frizzled-Arrow and NFull-PKC activities, respectively, that synergistically block Shaggy/GSK3 kinase and NICD activities to promote the LTM-related nuclear activities of Armadillo/β-Catenin and CREB (**Figure 2**). Blocking NICD activity might be important as our data from embryos suggest that this activity would suppress the expression of genes whose functions are promoted by NFull-PKC activity (Wesley et al., 2011). The Wingless/Wnt pathway is known to regulate many cytoskeletal remodeling processes during development (Payre et al., 1999; Delon et al., 2003; Chanut-Delalande et al., 2006). Thus, the simultaneous activation of this pathway and the Notch-PKC activity might be important for integrating signaling with F-actin-based processes during memory formation.

## FUTURE DIRECTIONS

One of the challenging questions is determining the cellular contexts for NFull-PKC and Wingless/Wnt activities in LTM formation. Do they function in response to neuron-neuron communication or neuron-glia communication? Do they function in the same cells? If not, how do they both promote LTM formation? This information would provide clues to the spatio-temporal configurations underlying LTM formation, as the two activities could regulate F-actin, hyper-PO4 CREB, and Armadillo/β-Catenin both spatially and temporally.

The more challenging question is how the ultradian oscillation of hyper-PO4 CREB is generated. We have some evidence from embryos and cultured cells that suggest the involvement of a self-sustaining mechanism. Immediately following NFull activation, when the PKC-dependent activity is high, P-Cactus and F-actin levels are high. These levels diminish over time, coincident with the accumulation of NICD (Wesley et al., 2011; Tremmel et al., 2013). Thus, it is possible that a single pulse of Notch activation generates the two Notch activities in a time sequence that leads to ultradian oscillation of hyper-PO4 CREB (**Figure 2B**). During vertebrate somitogenesis, both Notch and Wingless/Wnt activities are reported to manifest ultradian oscillation (Jensen et al., 2010; Kageyama et al., 2010). It would be fascinating to find out if Wingless/Wnt activity also oscillates during LTM formation. If it does, finding out whether it is in phase or out of phase with hypr-PO4 CREB oscillation would provide important clues for identifying the parameters controlling the oscillations. A mathematical analyzes of memory formation may become possible.

## IMPLICATIONS FOR DEMENTIA

The functions of the *Notch*, *PKC*, and *CREB* genes are disrupted in many neurodegenerative diseases, including Alzheimer’s disease (AD; Wang et al., 1994; Pakaski et al., 2002; Costa et al., 2003; Selkoe and Kopan, 2003; Pugazhenthi et al., 2011). One study has reported that NICD production is dramatically increased in the brains of AD patients (Berezovska et al., 1998). Furthermore, PKC and CREB activities are down regulated in AD animal models and the activation of a novel isoform of PKC or an increase in CREB phosphorylation is shown to significantly improve their cognitive function (Gong et al., 2004; Hongpaisan et al., 2011). Thus, there is a good chance that a disruption of mammalian non-canonical Notch-PKC-like activity is involved in dementia. That such an activity could also regulate F-actin is significant because studies in mice show that F-actin up regulation is important for memory formation and triggers the translocation of cytoskeleton-associated protein Arc/Arg3.1 into synapses (Lamprecht, 2011; Liu et al., 2012). Interestingly, Arc/Arg3.1 is required for proteolytic processing of Notch and synaptic plasticity (Alberi et al., 2011), which is consistent with our perspective that the sequence of activation of non-canonical and canonical Notch signaling might be important for LTM formation. Constitutive over-expression of either one of these activities might interfere with LTM formation. Since there is also evidence suggesting that the loss of Wingless/Wnt signaling is involved in AD (Boonen et al., 2009; Lucas et al., 2001; Jackson et al., 2002; Sofola et al., 2010), understanding how the non-canonical NFull–PKC signaling, the canonical Notch signaling, and Wingless/Wnt signaling function together in LTM formation might help us understand memory formation and memory loss upon neurodegeneration. It might also help us understand memory decline with age. One interesting possibility could be that the ultradian oscillation of hyper-PO4 CREB becomes more variable, due to either loss of synchrony between the oscillations in different cell types or deterioration in the coupling between the mechanisms controlling the frequency and the periodicity of the oscillation.

## Conflict of Interest Statement

The authors declare that the research was conducted in the absence of any commercial or financial relationships that could be construed as a potential conflict of interest.
